# Supplementary material for: ErbB3 Phosphorylation as Central Event in Adaptive Resistance to Targeted Therapy in Metastatic Melanoma: Early Detection in CTCs during Therapy and Insights into Regulation by Autocrine Neuregulin
Source: Cancers (Basel). 2019 Sep 25;11(10):1425. doi: 10.3390/cancers11101425 (PMC6826737; doi:10.3390/cancers11101425)
Supplement: Supplementary file 1 [file cancers-11-01425-s001.zip › cancers-580547-suppl-final2.docx]

Supplementary Materials: ErbB3 Phosphorylation as Central Event in Adaptive Resistance to Targeted Therapy in Metastatic Melanoma: Early Detection in CTCs During Therapy and Insights into Regulation by Autocrine Neuregulin

Ciro Francesco Ruggiero, Debora Malpicci, Luigi Fattore, Gabriele Madonna, Vito Vanella, Domenico Mallardo, Domenico Liguoro, Valentina Salvati, Mariaelena Capone, Barbara Bedogni, Paolo Antonio Ascierto, Rita Mancini and Gennaro Ciliberto


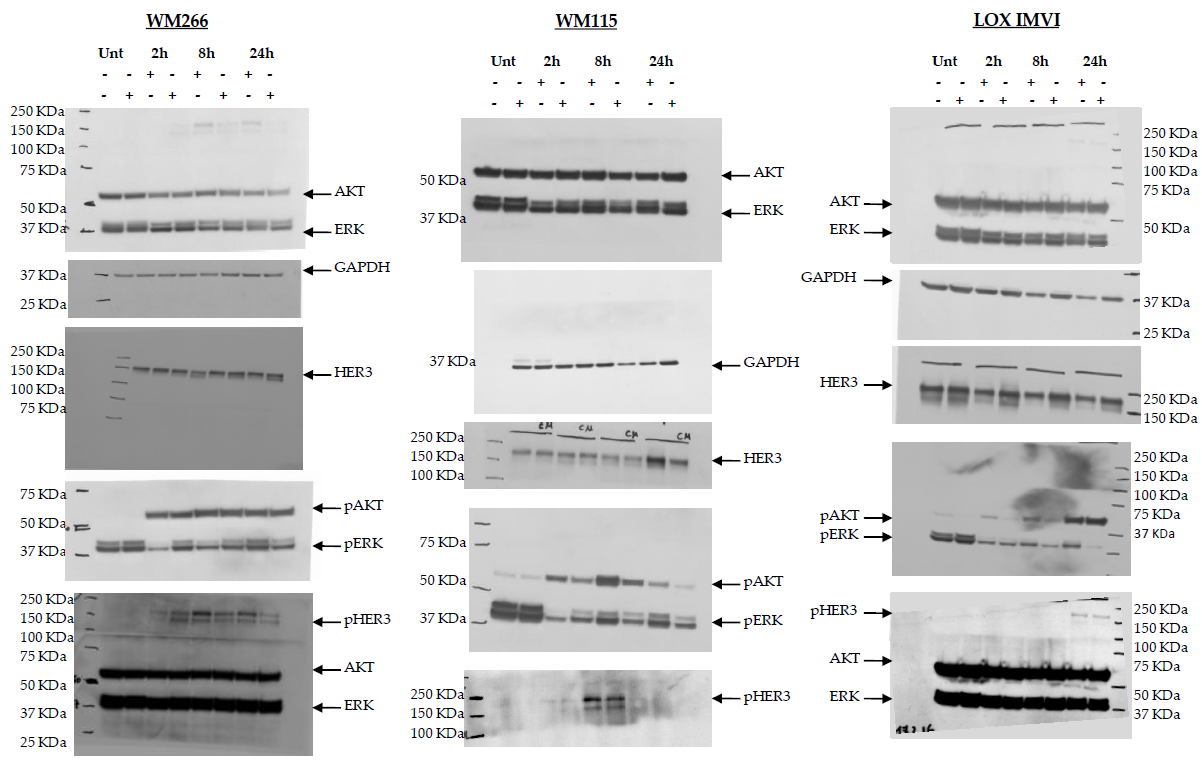


**Figure S1.** Whole blots of Figure 1B.


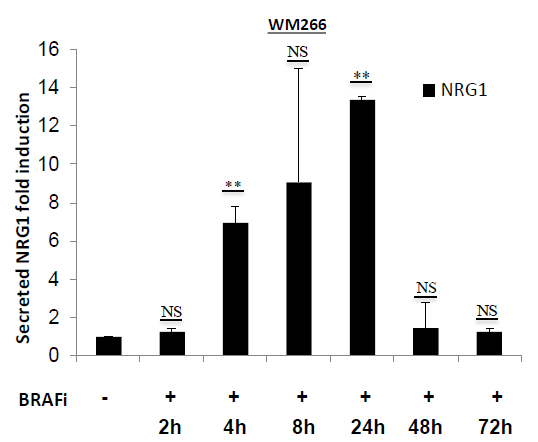


**Figure S2.** Increased levels of NRG-1 are released in cell media upon short term BRAF inhibitor treatment in WM266 melanoma cells. WM266 melanoma cell lines were starved and then treated with BRAFi (0.5 μM) for different times (from 2 h to 72 h). Cell media was collected and analyzed by immunoassay using the NRG-1 capture antibody. *: p < 0.05, **: p < 0.01, NS: p > 0.05.


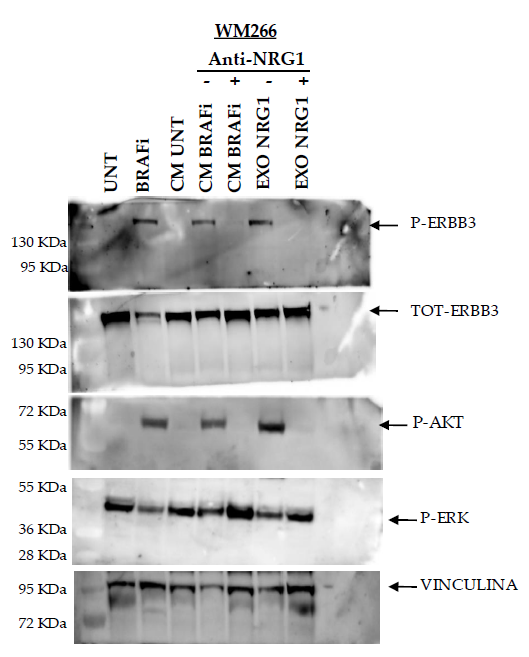


**Figure S3.** Whole blots of Figure 2B.


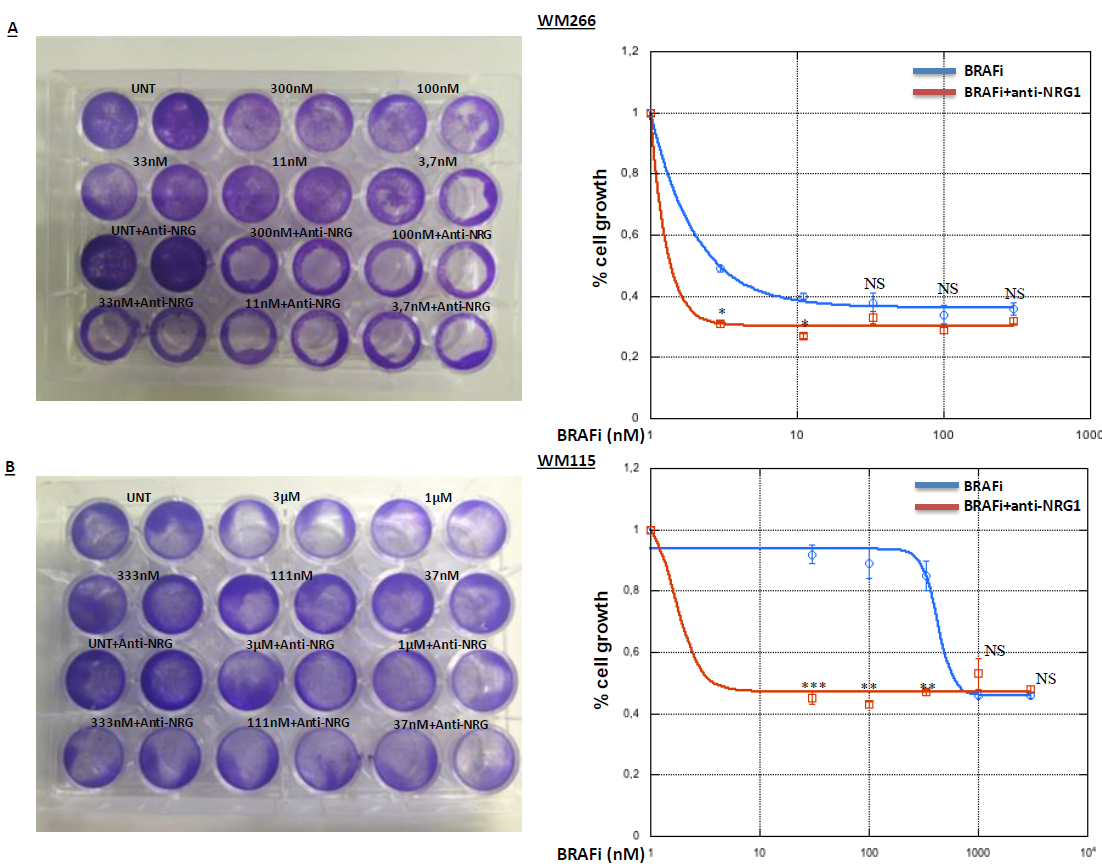


**Figure S4.** (**A**) Colony formation assay (left panel) shows that co-treating BRAF inhibitor with Anti-NRG improves the inhibition of cell growth compared to BRAFi alone. In WM266 melanoma cells. In the right panel the quantitative results by KaleidaGraph software are reported. (**B**) The same result has been confermed also in WM115 cells.


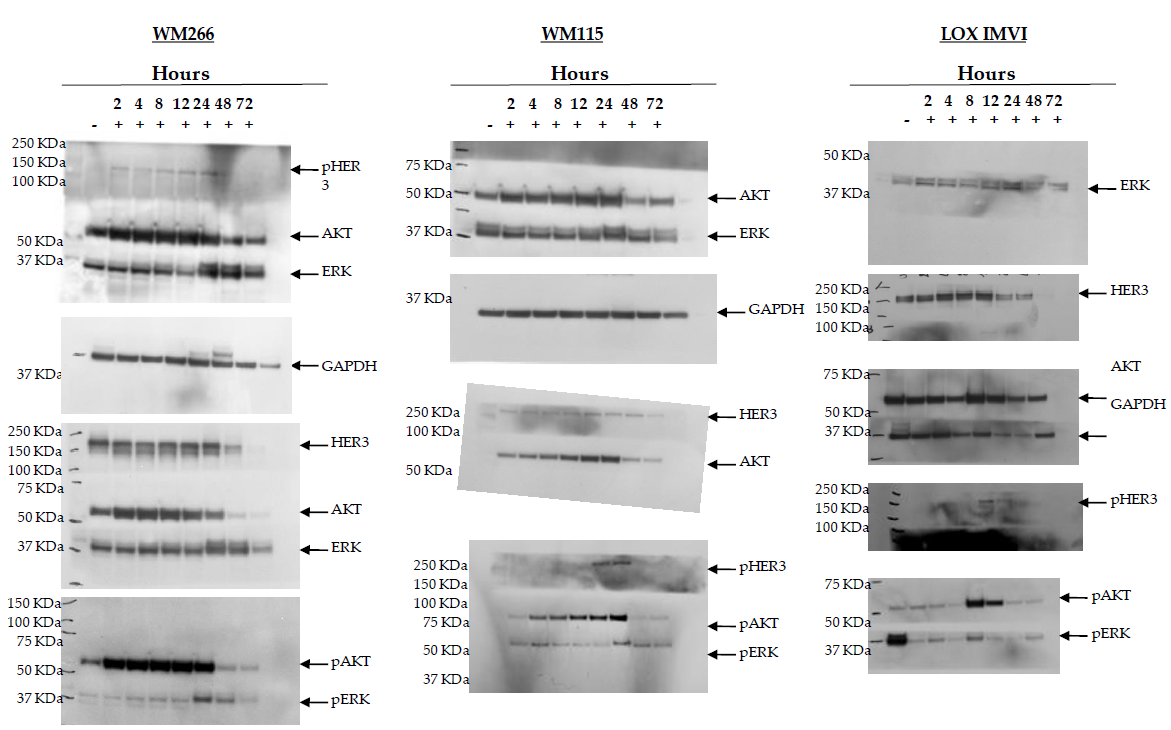


**Figure S5.** Whole blots of Figure 3A.


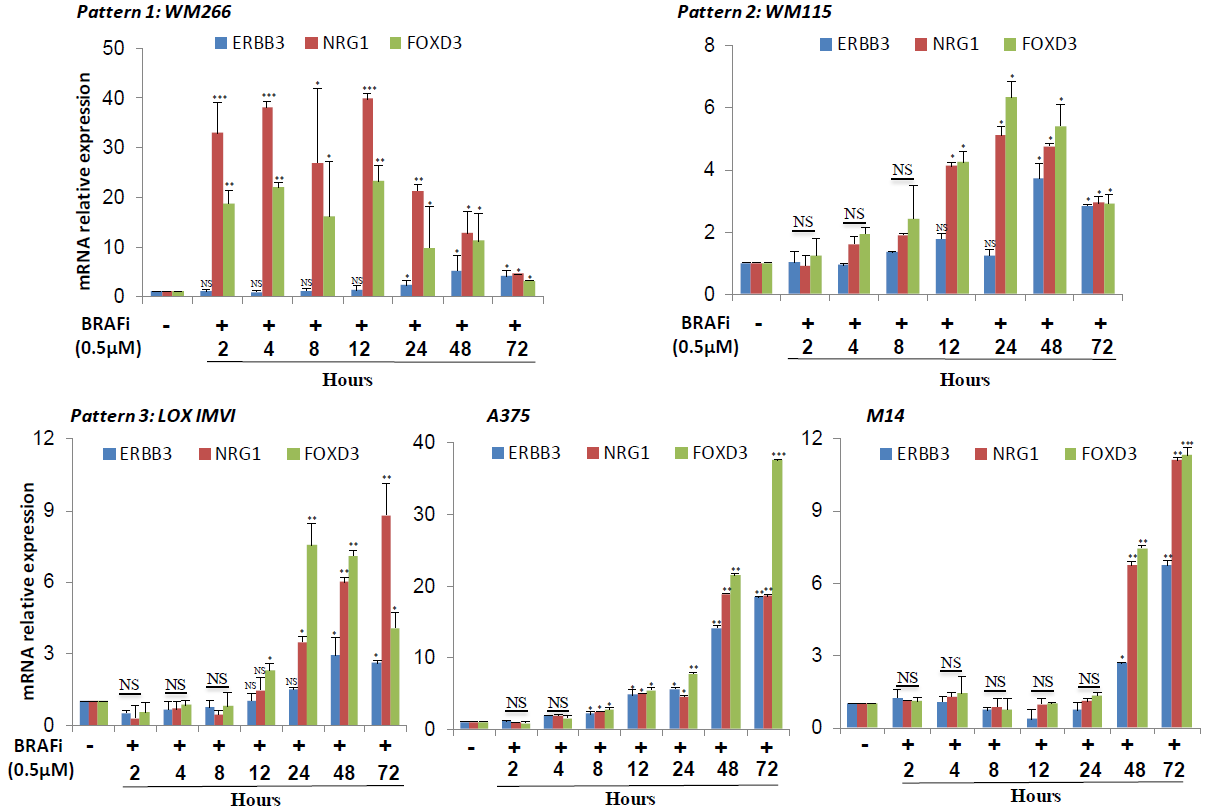


**Figure S6.** Melanoma cell lines (WM266, WM115, LOX IMVI, A375 and M14) were starved and then treated with BRAFi (0.5 μM) for different times (from 2 h to 72 h). Total RNA was extracted and subjected to qRT-PCR in order to evaluate the kinetic activation of NRG1, ErbB3 and FOXD3 genes. GAPDH reference gene was used for normalization. *: *p* < 0.05, **: *p* < 0.01, ***: *p* < 0.001, NS: p > 0.05.


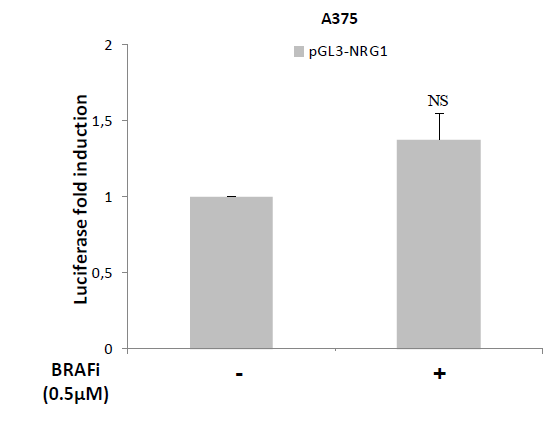


**Figure S7.** Results show that no luciferase induction occurred after BRAFi treatment in A375 melanoma cells. NS: p > 0.05.


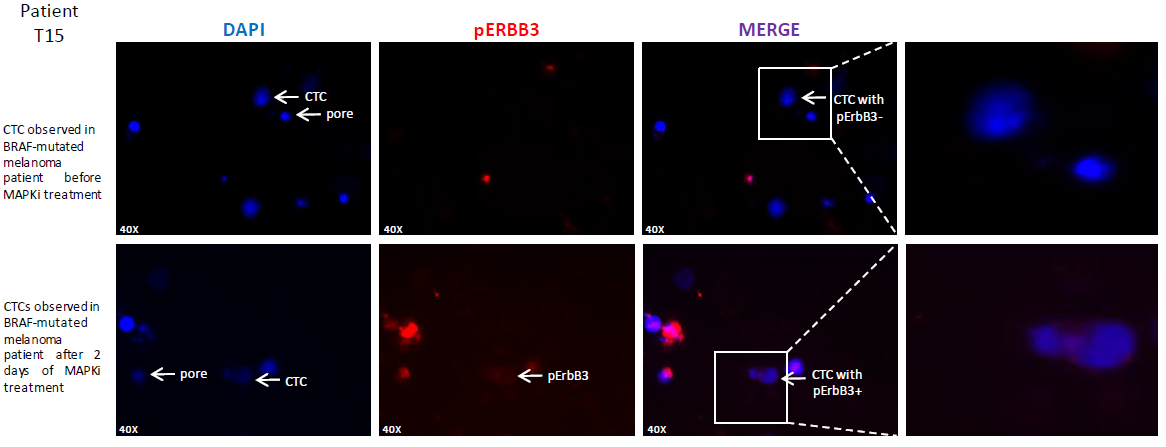


**Figure S8.** CTCs isolated by CELLSEARCH® Circulating Tumor Cell Kit are subjected to Immunofluorescence analysis in order to evaluate pErbB3 activation through pimary pErbB3 antibody and secondary Anti-Texas Red Antibody. Results show that in T15 BRAF-mutated melanoma patient the activation of phopho-ErbB3 was observed in circulating tumor cells upon 2 days of MAPKi treatment (Dapi = CTCs nuclei; Red = pErbB3).


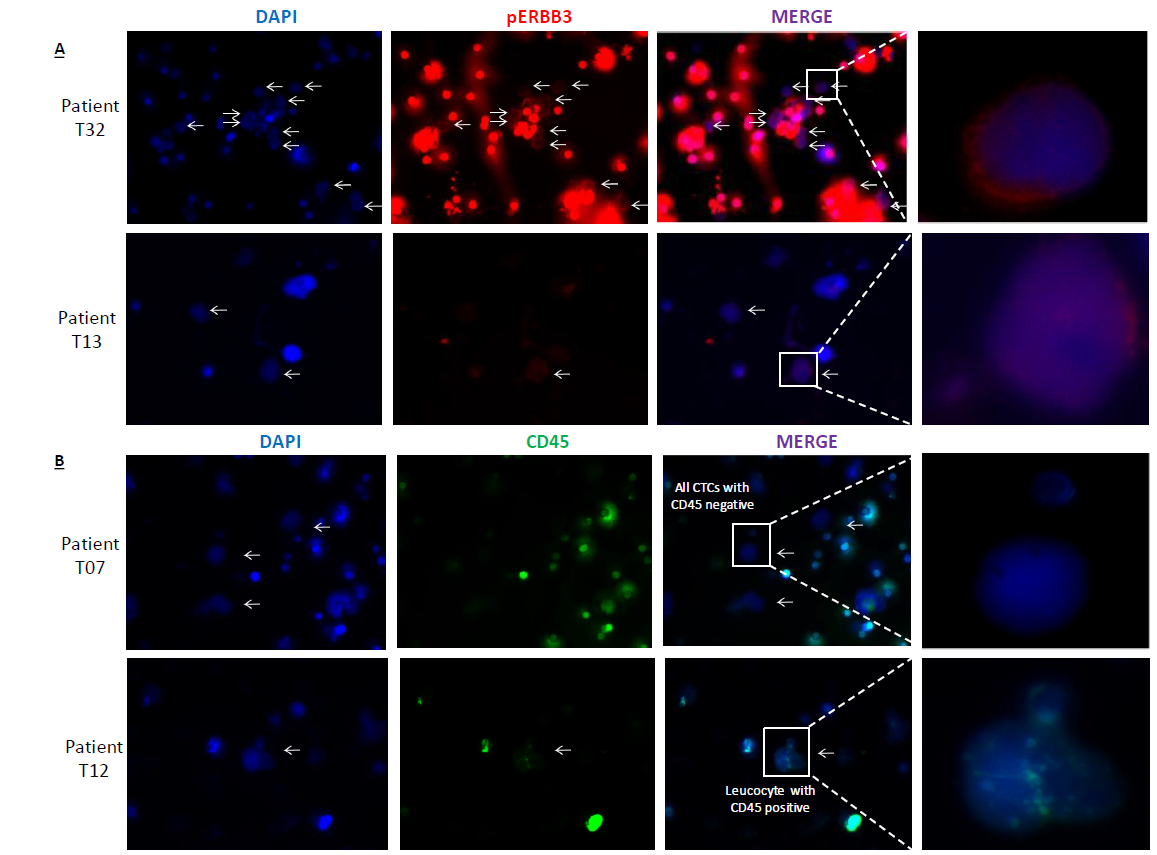


**Figure S9.** (**A**) Immunofluorescence analyses show the activation of pErbB3 detected in two melanoma patients after short term MAPKi treatment. The white arrows indicate CTCs in the pictures. (**B**) Positive signal for CD45 (green color) is evident in leucocyte (white arrow of patient T12) but not in CTCs as observed in patient T07. Scale bars: 50 μm.

| 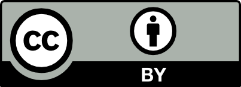 | © 2019 by the authors. Licensee MDPI, Basel, Switzerland. This article is an open access article distributed under the terms and conditions of the Creative Commons Attribution (CC BY) license (http://creativecommons.org/licenses/by/4.0/). |
| --- | --- |
